# The Role of Technology and the Continuum of Care for Youth Suicidality: Systematic Review

**DOI:** 10.2196/18672

**Published:** 2020-10-09

**Authors:** Hannah Szlyk, Jia Tan

**Affiliations:** 1 School of Social Work Rutgers, The State University of New Jersey New Brunswick, NJ United States; 2 The Brown School Washington University in St Louis Saint Louis, MO United States

**Keywords:** youth, suicide prevention, technology, continuum of care

## Abstract

**Background:**

Youth suicide is a global public health issue, and using technology is one strategy to increase participation in preventive interventions. However, there is minimal knowledge on how technology-enhanced interventions for youth correspond to the stages of care, from illness or risk recognition to treatment follow-up.

**Objective:**

This systematic review aims to examine the efficacy of technology-enhanced youth suicide prevention and interventions across the continuum of care.

**Methods:**

Four electronic databases were searched up to spring 2019 for youth suicide preventive interventions that used technology. The review was not restricted by study design and eligible studies could report outcomes on suicidality or related behaviors, such as formal treatment initiation. An adapted version of the Methodological Quality Ratings Scale was used to assess study quality.

**Results:**

A total of 26 studies were identified. The findings support the emerging efficacy of technology-enhanced interventions, including a decline in suicidality and an increase in proactive behaviors. However, evidence suggests that there are gaps in the continuum of care and recent study samples do not represent the diverse identities of vulnerable youth.

**Conclusions:**

The majority of identified studies were conducted in school settings and were universal interventions that aligned with the illness and risk recognition and help-seeking stages of the continuum of care. This field could be strengthened by having future studies target the stages of assessment and treatment initiation, include diverse youth demographics, and examine the varying roles of providers and technological components in emerging interventions.

## Introduction

### Background

Youth suicide is a global public health crisis. In the United States, suicide is the second leading cause of death for children and youth aged 10-24 years [1]. Globally, suicide is the third leading cause of death for youths aged 15-19 years [2]. In addition to deaths by suicide, suicidality includes suicidal ideation and related behaviors, such as plans to attempt suicide and actual suicide attempts [3]. Thus, research suggests that the risk of youth suicide may even be more pervasive as many youths experience suicidal ideation and nonfatal suicidal behaviors [4]. Universally, adolescence and early adulthood are vulnerable periods for when suicide risk is particularly elevated [5].

Therefore, it is important that youths have access to global systems of mental health care. Mental health services and resources may range from promoting illness recognition (in the case of suicidality, this may include risk factor recognition) to providing targeted treatment and to offering follow-up services. This range, which spans the levels of intensity in care, is often called the *continuum of care* [6,7]. Suicidal individuals who are engaged in an integrated continuum of mental health care may experience decreases in suicidality [8]. However, youth engagement in the continuum of mental health care is often complicated as a consequence of developmental changes, the delayed detection of symptoms, and delayed access to treatment [9]. Thus, researchers need to ensure that available interventions are tailored to specifically meet youths’ needs and correspond with the stages of the continuum of care [9].

Technology is one of the identified mediums to bridge gaps in the continuum of suicide preventive interventions [10]. Technology is especially relevant to engaging youths around the world. Research suggests that most young people in the United States and in developed countries have access to smartphones [11,12], whereas access increases among younger cohorts of emerging economies [12]. Therefore, the use of technology may address barriers to face-to-face care, such as access, reach, and stigma [10,13]. *Technology-enhanced interventions* use technology to solely deliver or serve as a component of an intervention and can include a mobile phone app, text messaging, telephone, videos, and web-based platforms [13]. Previous reviews on this topic have been restricted to gatekeeper interventions [14], including interventions across the lifespan or interventions designed to address broad mental health issues [15], focused on specific technologies [16], or that may be outdated as new interventions have since been developed [17]. No known review has explored how current technology-enhanced suicide interventions for youth correspond to the stages of the continuum of care. Thus, there is an incomplete understanding of the breadth and efficacy of preventive interventions that use technology and serve youth at risk of suicide.

### Objectives

To address these gaps in the literature, this systematic review aims to examine the efficacy of technology-enhanced youth suicide prevention and interventions across the continuum of care. The authors evaluated study outcomes in addition to suicidality, including help-seeking behaviors and coping skills, to better assess how the literature supports youth in leading lives worth living. The findings have implications for how suicidology may address identified gaps in the stages of the continuum of technology-enhanced suicide interventions and enhance care for vulnerable youths.

## Methods

### Search Strategy

**Figure 1:**
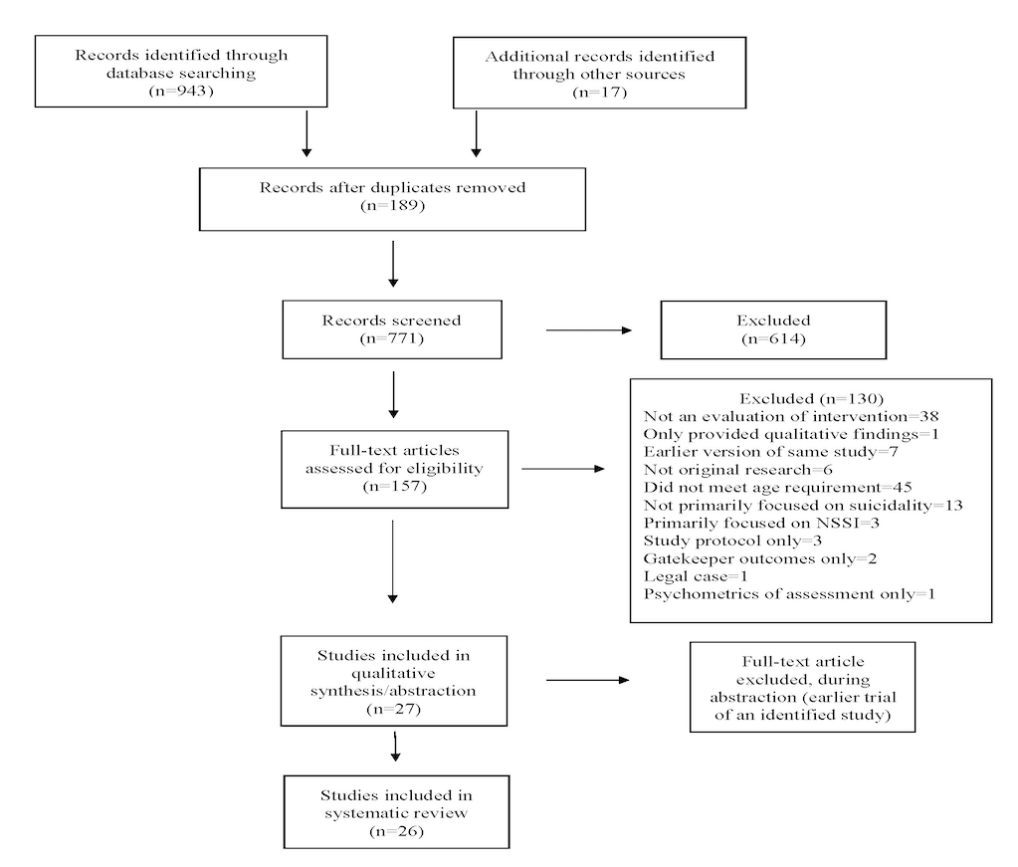
PRISMA (Preferred Reporting Items for Systematic Reviews and Meta-Analyses) diagram for search strategy.

The search was conducted in spring 2019, and the systematic review adhered to the PRISMA (Preferred Reporting Items for Systematic Reviews and Meta-Analyses) guidelines (Figure 1) [18].

Electronic databases were searched (PsycINFO, CINAHL [Cumulated Index to Nursing and Allied Health Literature], Ovid MEDLINE, ClinicalTrials.gov) using search terms specific to youth, suicidality, technology, and interventions (Multimedia Appendix 1). The study selection and data extraction were conducted by 2 assessors (the first and second authors). Eligible studies were required to focus on youths who were at potential risk, at high risk, or struggling with suicidality, be in English, and have the majority of participants between the ages of 12 and 24 years [19,20]. Selected studies adhered to the definition of technology-enhanced interventions (as previously defined) by Kreuze et al [13]. The investigators decided not to exclude studies that had primary outcome variables other than suicidality. This decision was based on a preliminary search of the literature, in which the investigators noticed that studies of technology-enhanced interventions for youth fell into several primary outcome domains (see the Data Extraction section for the outcome domains explored). The intention was to identify studies that may have been overlooked in previous reviews restricted to outcomes of suicidality and that applied to the stages of the continuum of care. Relevant systematic reviews were also cross-checked to adjust initial search terms and to potentially identify studies that had been missed in the final search.

Studies that focused on assisted suicide, nonsuicidal self-injury, postvention, or only gatekeeper outcomes were excluded from the final sample. In addition, the investigators excluded studies that only used technology to collect information about participant characteristics and behaviors, which only provided qualitative results, or only discussed the psychometrics of their assessment tool. No exclusion was placed on the trial design. When multiple publications of the same intervention were identified, the most recent or the most advanced trial was selected. Preliminary searches were organized using Endnote [21], and screening and data extraction were conducted using Covidence [22] and spreadsheets.

### Data Extraction

Data were extracted for study characteristics (Tables 1 and 2). As gender bias in peer review and in first author publications exists [23,24], the first author’s gender identity was extracted and determined by searching department profiles. In addition, as suicidology is considered a multidisciplinary field, the investigators decided to document the first author’s field of degree by searching web-based profiles and curriculum vitae. Studies’ stages on the continuum of care were also extracted, and studies could cover more than 1 stage. The continuum of care framework used in this review was based on the literature from various health disciplines [25-27] and included the following stages: illness or suicide risk factor recognition, help seeking, assessment, treatment initiation, treatment module, treatment engagement, and follow-up.

Quality of study design and methodology were assessed using an adapted version of the Methodological Quality Rating Scale (MQRS; see Multimedia Appendix 1 for full scale) [28]. The MQRS was originally developed to review alcohol treatment outcomes and covered 12 domains, such as study design, documentation of quality control of treatments, and reports of participants lost to attrition [28,29]. Each domain is rated by the study’s strategies to decrease bias, with studies reporting more rigorous strategies receiving a higher quality score. The MQRS has been used to review other substance and mental health intervention studies [30,31] and has been adapted to evaluate the inclusion of theoretical frameworks and cultural linguistic adaptions in study development [32,33]. The investigators assessed interrater reliability by calculating the percent agreement for each MQRS domain. For example, there was an 81% agreement on both investigators’ ratings by the domain of study design (see Multimedia Appendix 1 for the percent agreement for all MQRS domains).

The outcome attainment of each selected study was evaluated using a categorization system for statistical significance and outcome direction [33]. Outcomes were grouped into the following domains: suicidality (ie, suicidal ideation, planning suicide attempts, and attempts), co-occurring mental health issues (ie, anxiety, depression), youth’s perceptions and knowledge about suicide, help-seeking behaviors, proactive coping behaviors, and formal treatment initiation, which is the official beginning of participation in outpatient or inpatient mental health treatment.

The investigators documented whether the outcomes related to these domains demonstrated statistically significant changes (at least *P*<.05) and whether the change occurred in the desired direction (ie, a decrease in suicidal ideation) [33]. Differences in coding and ratings during the phases of study screening and data extraction were resolved through consensus between the 2 investigators.

## Results

### Study Characteristics

The final sample included 26 studies (Table 1) [34-59]. Although various professional disciplines were represented (ie, social work, medicine, public health), over half (n=16) of the first authors held a doctoral degree in psychology. In total, 16 of the first authors were female; 13 studies were conducted in the United States. Among studies that provided participant ages, the mean or median age ranged from 14.7 to 23 years. Female participants represented the majority of the sample participants. Of the studies that did report youth ethnicity, the majority of participants identified as White; no study reported the sexual or gendered minority identity of the participants. A total of 13 studies were conducted in an educational setting.

The selected studies included a variety of preventive interventions (Table 2); the SOS (Signs of Suicide) model was included in 2 studies: the original model for high schools [34] and the adapted model for middle schools [41]. The modalities of technology most often used were phones and web or web-based platforms. Half of the studies (n=13) described indicated interventions for youth suicidality. A total of 12 studies were randomized controlled trials (RCTs). The outcomes of the adapted MQRS tool ranged from 0 (lowest quality) to 18 (highest quality), with an average score of 10.4 and range of 3 to 16.

**Table 1:** Study characteristics.

Authors (reference)		First author’s field		Country		Setting		Sample size, n		Age (years), mean (SD)^a^		Majority gender of sample		Majority ethnicity of sample			
**Universal**																	
	Aseltine et al [34]		Sociology		United States		High school		2100		Not available		Female		Hispanic, non-White		
	Bailey et al [35]		Behavioral health sciences		Australia		High school		129		16.7 (N/A^b^)		Male		White		
	Freedenthal [36]		Social work		United States		High school		146		15.8 (1.2)		Female		Hispanic, non-White		
	Haas et al [37]		Psychology		United States		University; web based		1162		Not available		Female		Not available		
	Han et al [38]		Public health		Australia; China		University; web based		257		Subsamples: 18.6 (1.02) 20.1 (2.08)		Female		Chinese		
	Pisani et al [39]		Psychology		United States		Rural high school		42		Not available		Female		White		
	Robinson et al [40]		Psychology		Australia		High school		69		16.4 (N/A)		Not available		Not available		
	Schilling et al [41]		Public health		United States		Middle school		386		Not available		Female		White		
	Wyman et al [42]		Psychology		United States		High school		2675		Not available		Female		White		
**Selective**																	
	Dickter et al [43]		Psychology		United States		Hospital; web based		83		17.5 (2.04)		Female		White		
	Hetrick et al [44]		Psychology		Australia		Community mental health clinic		101		18.7 (2.8)		Female		Not available		
	Iorfino et al [45]		Psychology		Australia		Primary mental health clinic; web based		232		20.4 (2.59)		Female		Not available		
	King et al [46]		Psychology		United States		University; web based		76		22.9 (5.0)		Female		White		
**Indicated**																	
	Bertolote et al [47]		Clinical sciences; psychiatry		Brazil, India, Sri Lanka, Iran, China		Emergency department		1867		23^c^ median age		Female		Indian		
	Czyz et al [48]		Psychology		United States		Hospital		36		15.4 (1.36)		Female		White		
	Hetrick et al [49]		Psychology		Australia		High school; web based		50		14.7 (1.4)		Female		Not available		
	King et al [50]		Psychology		United States		Hospital		448		15.6 (1.31)		Female		White		
	King et al [51]		Psychology		Australia		Hotline or counseling center		101		Not available		Female		Not available		
	Mehlum et al [52]		Medicine or psychology		Norway		Child and adolescent psychiatric outpatient		77		15.6 (1.5)		Female		Norwegian		
	Normand et al [53]		Psychiatry		France		Hospital		173		Subsamples: 17.9 (1.9) 18.4 (1.8)		Female		Not available		
	O’Brien et al [54]		Social work		United States		Psychiatric outpatient		20		15.7 (1.6)		Female		White		
	Rosenbaum et al [55]		Psychiatry		United States		Emergency department		181		14.7 (2.0)		Female		Hispanic, non-White		
	Tan et al [56]		Psychology		China		Web based		725		21.2 (3.69)		Female		Not available		
	Yen et al [57]		Psychology		United States		Psychiatry inpatient unit		20		15.9 (1.5)		Female		White		
	Yen et al [58]		Psychology		United States		Psychiatry inpatient unit		50		15.7 (1.53		Female		White		
**All tiers**																	
	Silverstone et al [59]		Psychiatry		Canada		Middle and high school		6651		Not available		Female		Not available		

^a^For mean age, full sample information has been provided; subsample information was reported when the mean and SD of the full sample could not be generated due to insufficient information.

^b^N/A: not applicable.

^c^Authors only provided median age across study sites.

**Table 2:** Study characteristics continued.

Authors (references)		Design		Intervention name		Description		Technology used		MQRS^a^ score (0-18)	
**Universal**											
	Aseltine et al [34]		RCT^b^		SOS^c^		Aims to raise awareness of suicide risk and promote help seeking for youth and peers. Curricula delivered via video and discussion. Participation in program over 2 days		Video		14
	Bailey et al [35]		Pre, posttest		safeTalk		A 1-time, 3-hour workshop delivered to students by a trainer and school staff. Uses presentations, video, discussion, questions, and role plays. Teaches students about suicide risk, perceptions about suicide, and help-seeking strategies		Video, phone		10
	Freedenthal [36]		Quasi-experimental trial		Yellow Ribbon		A 60-min student leadership training for students and school staff conducted by program trainers. Training is focused on warnings signs of suicide among youth, myths about suicide, and the importance of seeking help for peers or for oneself. Content is delivered via a slide show and Ask4Help cards		Digital slide presentation		11
	Haas et al [37]		Prospective cohort study		College screening		An interactive, web-based program for university students officially called the College Screening Project. The web-based screening identified at-risk students, supported them in getting help, and helped to determine the proportion of students who entered treatment		Web-based screener, email, web-based chat		9
	Han et al [38]		RCT		ProHelp		A brief, 2-module web-based psychoeducational program that aims to teach students about risk factors for suicide, stigmatizing attitudes, and barriers to help seeking		Web-based platform		12
	Pisani et al [39]		Field test		Text4Strength		An automated, interactive text messaging intervention developed for early adolescents in rural communities. It is an extension of the Sources of Strengths program. Youth received messages over 9 weeks that were related to topics of emotion regulation, social connections, and help seeking		Video, text messaging		11
	Robinson et al [40]		Pilot study		Social Media Message		A social media message intervention that was developed by youth for at-risk peers. Designed to increase youth awareness about suicide, risk factors, and strategies to help peers and themselves. Participants evaluated 2 social media messages		Video, phone, tables and computers, web based		3
	Schilling et al [41]		RCT		SOS-Middle School		Similar to the SOS high school version. Features a 17-min DVD that includes 3 age-appropriate vignettes; a group discussion by middle school students about depression, suicide, bullying, self-injury, and getting help; and a student interview with a school-based counselor to model getting help. Delivered by trained school personnel		DVD or video		13
	Wyman et al [42]		RCT		Sources of Strength		Aims to improve youth help-seeking behaviors and proactive coping to reduce the risk of suicide. The program has 3 standard phases: (1) school and community preparation, (2) peer leader training, and (3) schoolwide messaging through video and text messaging. Premise is that peer and staff training (varying from 1 to 6 hours) in curriculum encourages sustainability of the program		Video, text messaging		11
**Selective**											
	Dickter et al [43]		Nonrandomized trial, 2 treatment groups		CATCH-IT: The Competent Adulthood Transition with Cognitive Behavioral and Interpersonal Training		The Competent Adulthood Transition with Cognitive Behavioral and Interpersonal Training consists of 14 self-guided, web-based modules that use techniques from CBT^d^ and interpersonal psychotherapy. Aims to teach skills for increasing resiliency against depressive disorders and suicidality		Web-based platform		9
	Hetrick et al [44]		Prospective cohort study		Monitoring Tool		A web-based tool for self-monitoring of depression and suicidal ideation that tracked changes in symptoms and alerted clinicians. Participating youth completed the tool between 2 and 8 times (duration varying between 4 and 83 days)		Web-based platform		10
	Iorfino et al [45]		Prospective cohort study		Synergy Online System		An initial clinical assessment on the web before a face-to-face or web-based clinical appointment. The initial clinical assessment assesses a range of mental health outcomes (14 modules, approximately 45 min to complete). At the end of the suicidality module, the algorithms assess current and past suicidality, which alerts clinical staff if the current suicide risk is high		Video, web-based platform		6
	King et al [46]		RCT		*E*Bridge		The Electronic Bridge to Mental Health Services is a web-based screening and intervention program for college students at risk for suicide. The program provides students with feedback from the screening and information about resources and can link students with web-based counseling services. The program aims to increase help-seeking and eventual use of mental health services. Length of program depended on level of student interaction		Web-based platform		11
**Indicated**											
	Bertolote et al [47]		RCT		BIC		A brief educational intervention with periodic follow-up contacts for suicide attempters conducted at global emergency departments and was part of the WHO^e^ Multisite Intervention Study on Suicidal Behaviors (SUPRE-MISS). The BIC procedure includes a standard 1-hour individual information session at the time of discharge. Follow-up contacts by health professionals were 1 week; 2, 4, 7, and 11 weeks; and 4, 6, 12, and 18 months after discharge		Phone		17
	Czyz et al [48]		RCT		MI-SafeCope		A motivational interview-enhanced safety planning intervention for teens hospitalized because of suicide risk. The intervention includes 3 components: an individual session, a family session, and a 2-week postdischarge booster call by phone (with the intervention counselor). Youth also provided assessments via text message at 1 and 3 months after discharge		Phone, text messaging		14
	Hetrick et al [49]		RCT		Reframe-IT		An internet-based CBT program that aims to reduce suicide-related behaviors, depression, anxiety, and hopelessness and improve problem solving and cognitive and behavioral issues. The intervention consisted of 8 modules of CBT delivered on the web over 10 weeks		Web-based platform		12
	King et al [50]		RCT		YST-II		The Youth-Nominated Support Team–Version II for suicidal adolescents provides psychoeducation and ongoing consultation for the parent-approved adult support persons that have been nominated by the adolescent. The support persons maintain regular supportive contact with the adolescents via phone for 3 months following hospitalization		Phone		14
	King et al [51]		Pre-post tests		Kids Help Line		Trained help line counselors assessed changes in suicidality and mood for youth callers at the beginning of the session and at the conclusion of the phone session. Mean duration of calls was 40 min (range 10-120 min)		Phone		4
	Mehlum et al [52]		RCT		DBT-A: Dialectical Behavior Therapy-Adolescent		Dialectical behavior therapy for adolescents lasts from 3 to 5 months, includes parents or other caregivers in weekly skills training groups, and has a skills module to support teens with emotion dysregulation and their families. This trial was delivered over 18 weeks and was delivered by trained mental health professionals. Coaching sessions were delivered over the phone		Phone		16
	Normand et al [53]		Prospective cohort study		4-Phone-Calls		Hospital staff called the youth 1 week, 1 month, 6 months, and 12 months after discharge for a suicide attempt. The interviews during the phone calls included informal and formal assessment of current symptoms and the youth’s safety		Phone		7
	O’Brien et al [54]		Pilot study		Crisis Care		A smartphone app intervention developed specifically for suicidal adolescents and their parents to use after discharge from the hospital. The app provides access to coping skills and immediate access to help, if needed		Smartphone app		4
	Rosenbaum et al [55]		RCT		FISP		The Family Intervention for Suicide Prevention is developed for youth admitted to emergency departments post–suicide attempt. Youth and their families participate in a CBT session aimed to increase motivation for follow-up treatment and safety postdischarge. Participants also received structured phone calls 48 hours and often 1, 2, and 4 weeks postdischarge to promote outpatient treatment attendance		Phone		11
	Tan et al [56]		Pilot study		Microblog Intervention		An intervention developed for users of the Sina Weibo microblogging platform. Participants received direct messages designed to respond to high suicide risk postings. The intervention aimed to increase help seeking for at-risk users and peers		Web-based platform; Sina Weibo		5
	Yen et al [57]		Pre-post test		STEP		Includes an in-person phase (4 sessions) and a remote delivery phase (text messaging and phone calls). The inpatient sessions focus on psychoeducation and coping skills. The remote delivery phase consists of weekly phone calls and daily text messages to provide skills practice reminders and to monitor mood		Phone, text messaging		12
	Yen et al [58]		RCT		CLASP-A		The Coping Long Term with Active Suicide Program for Adolescents program is adapted for adolescents hospitalized for suicidal ideation or a suicide attempt. The program includes 3 individual sessions and 1 family session and a series of outpatient phone calls to adolescent and a designated parent or guardian over 6 months of follow-up postdischarge		Phone		15
**All tiers**											
	Silverstone et al [59]		Pre-post and follow-up		EMPATHY		The multimodal program includes repeated data collection, identification of a high-risk group, a rapid intervention for the high-risk group (a supervised web-based CBT program), a universal CBT intervention, interactions with trained staff, and referrals to external medical and psychiatric services		Web-based platform		9

^a^MQRS: Methodological Quality Rating Scale.

^b^RCT: randomized controlled trial.

^c^SOS: Signs of Suicide.

^d^CBT: cognitive behavioral therapy.

^e^WHO: World Health Organization.

Regarding the continuum of care (Table 3), most studies were targeted to increase illness or risk factor recognition (n=11), to increase help seeking (n=10), and to guide youths through a treatment module (n=10; Table 2). The majority of studies (n=18) addressed >1 stage of the continuum of care. For example, 8 of the 9 universal interventions addressed illness or risk recognition and help seeking; 5 of the 11 indicated interventions focused on the stages (at least) of participating in the treatment module and treatment engagement. As illustrated in Table 4, among the 6 common outcome domains measured, most studies (n=22) reported suicidality (ie, ideation, attempts) as an important study variable, followed by co-occurring mental health issues (n=12; ie, depression, distress, or anxiety).

**Table 3:** Studies and their stages on the continuum of care.

Authors (references)		Illness or risk recognition	Help seeking	Assessment	Treatment initiation	Treatment module	Treatment engagement	Follow-up	
**Universal**									
	Aseltine et al [34]	✓^a^	✓	N/A^b^	N/A	N/A	N/A	N/A	
	Bailey et al [35]	✓	✓	N/A	N/A	N/A	N/A	N/A	
	Freedenthal [36]	✓	✓	N/A	N/A	N/A	N/A	N/A	
	Haas et al [37]	N/A	N/A	✓	✓	N/A	N/A	N/A	
	Han et al [38]	✓	✓	N/A	N/A	N/A	N/A	N/A	
	Pisani et al [39]	✓	✓	N/A	N/A	N/A	N/A	N/A	
	Robinson et al [40]	✓	✓	N/A	N/A	N/A	N/A	N/A	
	Schilling et al [41]	✓	✓	N/A	N/A	N/A	N/A	N/A	
	Wyman et al [42]	✓	✓	N/A	N/A	N/A	N/A	N/A	
**Selective**									
	Dickter et al [43]	N/A	N/A	N/A	N/A	✓	N/A	N/A	
	Hetrick et al [44]	N/A	N/A	✓	N/A	N/A	N/A	N/A	
	Iorfino et al [45]	N/A	N/A	✓	N/A	N/A	N/A	N/A	
	King et al [46]	✓	✓	N/A	✓	✓	✓	N/A	
**Indicated**									
	Bertolote et al [47]	N/A	N/A	N/A	N/A	✓	N/A	✓	
	Czyz et al [48]	N/A	N/A	N/A	N/A	✓	✓	✓	
	Hetrick et al [49]	N/A	N/A	N/A	N/A	✓	✓	N/A	
	King et al [50]	N/A	N/A	N/A	N/A	N/A	N/A	✓	
	King et al [51]	✓	N/A	N/A	N/A	N/A	N/A	N/A	
	Mehlum et al [52]	N/A	N/A	N/A	N/A	✓	✓	N/A	
	Normand et al [53]	N/A	N/A	N/A	N/A	N/A	N/A	✓	
	O’Brien et al [54]	N/A	N/A	N/A	N/A	✓	N/A	N/A	
	Rosenbaum et al [55]	N/A	N/A	N/A	N/A	N/A	✓	✓	
	Tan et al [56]	N/A	✓	N/A	N/A	N/A	N/A	N/A	
	Yen et al [57]	N/A	N/A	N/A	N/A	✓	✓	✓	
	Yen et al [58]	N/A	N/A	N/A	N/A	✓	✓	✓	
**All tiers**									
	Silverstone et al [59]	✓	N/A	✓	✓	✓	N/A	N/A	
Total, n		11	10	4	3	10	7	7	

^a^The study addresses that stage of the continuum.

^b^N/A: not applicable.

**Table 4:** Measured and significant intervention outcomes.

Authors (references)		Suicidality		Co-occurring mental health issues		Perceptions and knowledge of suicide		Help seeking		Coping behavior		Treatment initiation		Significant outcomes	
**Universal**															
	Aseltine et al^a^ [34]		✓^b^		N/A^c^		✓		✓		N/A		N/A		Suicide attempts, decrease; adaptive attitudes about suicide, increase; knowledge about suicide, increase
	Bailey et al [35]		✓		✓		✓		✓		N/A		N/A		Distress, decrease at T1^d^ and T2^d^; adaptive attitudes about suicide, increase; knowledge about suicide increase; help seeking for self at T2 and T3^d^, increase
	Freedenthal [36]		✓		N/A		N/A		✓		N/A		N/A		Help seeking by hotline, increase; help seeking from adult, decrease; help seeking from a peer, decrease
	Haas et al [37]		N/A		✓		N/A		N/A		N/A		✓		Outpatient treatment initiation for students who received evaluation and dialogue with counselor, increase
	Han et al^a^ [38]		N/A		N/A		✓		✓		✓		N/A		Knowledge of suicide, increase at posttest; attitude about help seeking from a professional, increase
	Pisani et al^a,e^ [39]		N/A		✓		✓		N/A		✓		N/A		Not reported
	Robinson et al [40]		✓		N/A		✓		✓		N/A		N/A		Not reported
	Schilling et al^a^ [41]		✓		N/A		✓		✓		N/A		N/A		Suicidal ideation, planning, or attempts decrease among intervention participants with pretest suicidal ideation; knowledge about suicide, increase
	Wyman et al^a^ [42]		✓		N/A		✓		✓		✓		N/A		Perceptions of seeking help from adults, increase; help seeking from nonmental health professional, increase; help seeking from a peer, increase
**Selective**															
	Dickter et al [43]		✓		✓		N/A		N/A		N/A		N/A		Suicidal ideation, decrease
	Hetrick et al [44]		✓		✓		N/A		N/A		N/A		N/A		Suicidal ideation, decrease; depression symptoms, significant decrease
	Iorfino et al [45]		✓		N/A		N/A		N/A		N/A		N/A		Not reported
	King et al^a^ [46]		✓		✓		✓		✓		N/A		✓		Stigma to seek help for mental health issues, decrease; help seeking from a mental health professional, increase; help seeking from family members, increase; help seeking from a peer, increase; outpatient treatment initiation, increase
**Indicated**															
	Bertolote et al^a^ [47]		✓		N/A		N/A		N/A		N/A		✓		Not reported
	Czyz et al^a^ [48]		✓		N/A		N/A		N/A		✓		N/A		Coping for suicidal behavior, increase; coping with safety plan, increase
	Hetrick et al^a^ [49]		✓		✓		N/A		N/A		✓		N/A		Not reported
	King et al^a^ [50]		✓		✓		N/A		N/A		N/A		N/A		Suicidal ideation, decrease at 6 weeks and 6 months of follow-up among multiple attempters
	King et al [51]		✓		✓		N/A		N/A		N/A		N/A		Suicidal ideation, decrease; distress, decrease
	Mehlum et al^a^ [52]		✓		✓		N/A		N/A		N/A		✓		Suicidal ideation, decrease; depression symptoms, decrease
	Normand et al [53]		✓		N/A		N/A		N/A		N/A		✓		Not reported
	O’Brien et al [54]		N/A		N/A		✓		N/A		N/A		N/A		Not reported
	Rosenbaum et al^a^ [55]		✓		✓		N/A		N/A		N/A		✓		Outpatient treatment initiation, increase
	Tan et al^a,^^e^ [56]		✓		N/A		N/A		N/A		N/A		N/A		Not reported
	Yen et al [57]		✓		N/A		N/A		N/A		N/A		✓		Suicidal ideation, decrease
	Yen et al [58]		✓		N/A		N/A		N/A		N/A		✓		Not reported
**All tiers**															
	Silverstone et al [59]		✓		✓		N/A		N/A		N/A		N/A		Suicidal ideation, planning, and attempts, decrease among actively suicidal participants; depression symptoms, decrease; anxiety, decrease

^a^Indicates the study was a randomized controlled trial.

^b^The study reports that outcome.

^c^N/A: not applicable.

^d^T1, T2, and T3 indicate the time trials of the larger study.

^e^The study’s purpose was to promote help seeking, but no variables directly reported the help-seeking behaviors of the participants.

In total, 9 of the 12 RCT studies and 8 studies of other designs reported significant changes in study outcomes. A total of 22 studies measured suicidality outcomes and only 9 of those studies reported significant changes (4 of which were RCTs). The next most common outcome domain measured was co-occurring mental health issues, and 5 of the 12 studies reported significant outcomes. In total, 6 of the 9 studies that measured perceptions and knowledge of suicide and 6 of the 8 studies that measured help-seeking behaviors noted significant findings. Finally, only 1 of the 5 studies that measured coping behaviors and 3 of the 8 studies that measured treatment initiation reported significant results.

## Discussion

### Principal Findings

The findings suggest that suicidologists around the world are working to utilize technology to prevent youth suicide. Results demonstrate that 17 interventions of varying study designs reported significant changes in at least one of the outcome domains. In addition, it is promising that the majority of selected studies were conducted in educational settings, which may increase opportunities for youth to learn about suicide risk, seek help, and participate in treatment beyond a formal, clinical setting. On the basis of the findings presented in Tables 1-3, these school-based and university-based interventions were mostly universal interventions and aligned with the illness and risk recognition and help-seeking stages of the continuum of care. In addition, these interventions mainly used videos and web-based platforms and predominantly demonstrated efficacy in increasing help-seeking behaviors and youth knowledge and perceptions about suicide.

However, the results from this review also illustrate that efforts are needed to test technology-enhanced interventions across the continuum of care. Among this review’s sample, few studies used technology to assess suicidality or to formally initiate mental health treatment. This gap in the continuum of care is crucial to address as these stages impact participation in treatment modules and, hopefully, prevent future deaths. During the initial search, the investigators noticed that many available electronic assessments were not specific to youth suicidality, had not been incorporated into an intervention trial, or were only in the early stages of development.

Regarding the other intervention tiers, only 4 studies, all using web-based platforms, were determined to be selective interventions and these studies spanned the stages of the continuum of care. Although 3 studies did note significant changes in multiple outcome domains, future research should focus on strategies to use technology to reach youths who are at higher risk of suicide across the continuum of care, as this appears to be an overlooked group in technology-enhanced interventions. It was also not surprising that the majority of indicated interventions addressed the treatment module, treatment engagement, and follow-up stages of care; these interventions also primarily used phones. Although several studies have reported significant improvements in youth suicidal behavior, future trials may extend treatment outcomes beyond suicidality to include coping behaviors (in this review, only 2 studies measured this) and other metrics that mark improvements in youth resiliency.

Although certain tiers of intervention and stages of the continuum of care were associated with specific types of technology, it may be premature to determine whether one modality is better associated with efficacy, acceptability, or feasibility. For example, it would be presumed that studies that use phones would achieve more successful metrics than a study that used a less-established or newer technology, such as a mobile phone app or web-based platform. However, this review suggests that studies with significant findings do not use one specific type of technology and therefore other factors, such as study design, intervention curriculum, and youth sample, may have a greater impact on an intervention’s success.

### Implications for Future Study Design

This review emphasizes that not all youth interventions that use technology are the same. Some interventions have more *human* and *face-to-face* involvement, whereas others are mainly automated or self-directed by the participant. *Behavioral intervention technology* (BIT) is a term used in other health disciplines to determine the level of human involvement and automation in an intervention [60]. In contrast, the suicidology literature most often evaluates the intervention as a whole and does not provide specific details about the technological components. Understanding the level of provider integration and the technological components of the BIT may also inform how interventions can be implemented in other settings and scaled to reach a broader youth consumer base [61]. For example, the review’s findings confirm that universal interventions that target help seeking, illnesses, and risk recognition are already tested on a larger scale versus selective and indicated interventions. Therefore, incorporating the BIT terminology and models may help suicidologists determine the efficacy and acceptability of specific intervention components, determine which technologies are better suited for specific stages of the continuum of care, and to disseminate other tiers of interventions to mental health systems that service at-risk and suicidal youth.

Scores on the MQRS demonstrated variability in the quality of the studies and may be a consequence of not restricting the study design for this systematic review. For example, several non-RCT studies did not have multiple sessions or have multisite trials for their intervention. However, many studies, regardless of study design, have underdeveloped MQRS domains in common, such as documenting the study’s theoretical foundation, conducting the study at more than 1 site, and reporting inclusion of a collateral data source. The limited inclusion of collateral data sources is problematic as it is considered best practice to supplement youth self-report with parent, guardian, or teacher observations in face-to-face interventions [62-65]. The issue of an incomplete client profile is most likely indicative of the challenges of data collection and digital interventions [66] and may be an issue that suicidologists implementing technology-enhanced interventions can collectively explore and tackle.

The scores on the MQRS also suggested that few studies adapted interventions to the cultural, social, and linguistic needs of their specific demographic. In addition, no study has reported the participants’ sexual identity, although suicide risk is particularly heightened among sexual minority youth [67]. As global research demonstrates that youth suicidality varies across age cohorts and demographics [5], technology-enhanced interventions need to mirror and be tailored for this diversity.

### Implications for the Profession of Suicidology

Diversity among suicide scholars and professional perspectives may also influence the impact that technology-enhanced interventions have on youths’ mental health [68-70]. It is promising that the first authors of 16 studies identified as female, considering the noted gender bias in peer review and grant funding [23,24,71]. This bias has been noted in suicidology as well, as the American Association of Suicidology has historically bestowed more men than women with its annual research awards [72]. In addition, doctoral training in psychology was most common among the first authors. Although this finding is not reflective of the potential professional diversity of the research team and of nonintervention studies, the subfield of technology-enhanced youth interventions may be mindful of how to mentor students and researchers from other disciplines to be principal investigators. Disciplines may include those who are involved in the frontline (such as nursing) or those who have extensive training in digital health literacy and computational methods (ie, health communication fields).

### Limitations

As the study sample was not restricted by research design, the investigators could not compare outcome effect sizes. Preliminary searches demonstrated that the pool of eligible studies that were also RCTs would be small and that the investigators did not want to overlook cutting-edge interventions that were in earlier stages of development. In addition, as this review was restricted to specific search guidelines and because suicide research is ever evolving, relevant studies may not have been included. For example, many studies were ineligible as they did not collect or report participants’ ages or validate that the participants were within the specific age range.

### Conclusions

This systematic review emphasizes the need for technology-enhanced interventions that extend beyond illness or risk recognition and help seeking, which are developed for diverse youth populations. Although technology shows promise in its utility to address suicidality and increase proactive behaviors, such as help seeking and coping skills, it is difficult to determine which types of technology are better associated with intervention efficacy, acceptability, and feasibility and better suited for specific stages of the continuum of care. The field of suicidology also faces challenges in capturing youth participants’ demographics on digital platforms, supplementing youth self-reports with collateral information, developing interventions suitable for underserved demographics, and involving researchers from diverse backgrounds and disciplines. Adoption of BIT terminology and frameworks may improve the understanding of both the roles of providers and technological components in technology-enhanced suicide preventive interventions for youth and how these interventions can be successfully implemented across the continuum of care and within mental health care systems.

## Appendix

Multimedia Appendix 1 Literature search terms and adapted study quality scoring sheet.

## Abbreviations

BIT: behavioral intervention technology
MQRS: Methodological Quality Rating Scale
PRISMA: Preferred Reporting Items for Systematic Reviews and Meta-Analyses
RCT: randomized controlled trial
