# Supplementary material for: The Role of Technology and the Continuum of Care for Youth Suicidality: Systematic Review
Source: J Med Internet Res. 2020 Oct 9;22(10):e18672. doi: 10.2196/18672 (PMC7584980; doi:10.2196/18672)
Supplement: Multimedia Appendix 1 [file jmir_v22i10e18672_app1.docx]

LITERATURE SEARCH TRACKING FORM

Ovid Medline

Date Searched: 4/5/19

Applied Database Supplied Limits: English

(exp Young Adult/ OR exp Adolescent/) OR (Adolescent* OR high school OR college OR university OR youth* OR Young adult* OR emerging adult* OR teen*).mp.) AND

(exp Hotlines/ OR exp Telemedicine/ OR exp Social Media/) OR (Teleconsultation OR telehealth OR telemedicine OR mhealth OR mobile health OR video conference* OR remote consultation* OR web 2 OR social media OR social medium OR twitter OR facebook OR Instagram OR tumblr OR reddit OR ebridge OR online OR electronic bridge).mp.

AND

(exp Suicide/ OR Suicid*.mp.)

NOT (systematic review OR newspaper OR meta analysis OR editorial OR book review).pt.

PsycINFO

Date Searched: 4/5/19

Applied Database Supplied Limits: excluded non-English and reports that were CEUs or magazine articles or electronic collections (e-resources)

Full Search Strategy:

DE "Suicide" OR DE "Attempted Suicide" OR DE "Suicidality" OR suicid*

AND

DE "Telemedicine" OR DE "Teleconferencing" OR DE "Online Therapy" OR DE "Teleconsultation" OR DE "Telepsychiatry" OR DE "Telepsychology" OR DE "Telerehabilitation" OR DE "Social Media" OR DE "Online Social Networks" OR Teleconsultation OR telehealth OR telemedicine OR mhealth OR “mobile health” OR “video conference*” OR “remote consultati*” OR “web 2.0” OR “social media” OR “social medium” OR “twitter” OR “facebook” OR “Instagram” OR “tumblr” OR “reddit” OR DE "Hot Line Services"

AND

DE "Colleges" OR DE "High Schools" OR DE "Emerging Adulthood" OR "adolescent" OR DE "Middle School Students" OR “young*”

CINAHL

Date Searched: 4/5/19

Applied Database Supplied Limits: excluded non-English and reports that were CEUs or magazine articles

Full Search Strategy:

((MH "Telemedicine+") OR (MH "Telepsychiatry") OR (MH "Text Messaging") OR (MH "Instant Messaging") OR (MH "Internet") OR (MH "Videoconferencing") OR (MH "Social Media") OR (MH "Online Services") OR (MH "Online Social Networking") OR ("online therapy") OR (MH "Telephone Information Services") OR ("hotline"))

AND

(MH "Suicide+" OR suicide* OR suicidal*)

("adolescent") OR (MH "Young Adult") OR (MH "Students, High School") OR (MH "Students, Middle School") OR (MH "Students, College") OR "youth" OR "young person" OR "teen*"

ClinicalTrials.gov

Date Searched: 4/8/19

Terms entered int Clinical Trials search engine. We completed 8 separate searches on this site, using the following terms:

Condition or Disease: Suicidal and Self-injurious Behavior AND Technology (other term)

Condition or Disease: Suicidal and Self-injurious Behavior AND mobile (other term)

Condition or Disease: Suicidal and Self-injurious Behavior AND online (other term)

Condition or Disease: Suicidal and Self-injurious Behavior AND hotline (other term)

Condition or Disease: Suicidal and Self-injurious Behavior AND text (other term)

Condition or Disease: Suicidal and Self-injurious Behavior AND video (other term)

Condition or Disease: Suicidal and Self-injurious Behavior AND tele (other term)

Condition or Disease: Suicidal and Self-injurious Behavior AND social media (other term)

|  | Adapted MQRS Survey for Continuum of Care and Technology-Enhanced Interventions Review |  |  |
| --- | --- | --- | --- |

|  | Domains |  | Rating | Inter-Rater Reliability ^a^ |
| --- | --- | --- | --- | --- |
| 1. | Study Design | 0 | Single group pretest-posttest | 0.81 |
|  |  | 1 | Quasi-experimental (nonequivalent control group/nonrandomization) |  |
|  |  | 2 | Randomization with control group |  |
|  |  |  |  |  |
| 2. | Theoretical Foundation | 0 | Treatment not theoretically based or not reported | 0.81 |
|  |  | 1 | Theoretical basis discussed |  |
|  |  |  |  |  |
| 3. | Quality Control | 0 | No intervention standardization specified | 0.73 |
|  |  | 1 | Intervention standardized by manual, procedures, specific training, etc. |  |
|  |  |  |  |  |
| 4. | Baseline | 0 | No baseline scores, participant characteristics or measures reported | 0.57 |
|  |  | 1 | Baseline scores, participant characteristics or measures reported |  |
|  |  |  |  |  |
| 5. | Measures | 0 | Reliability and validity of measures not reported or inadequate | 0.53 |
|  |  | 1 | Reported reliability and validity of measures adequate |  |
|  |  |  |  |  |
| 6. | Follow-up Length | 0 | No follow-up reported | 0.76 |
|  |  | 1 | Follow-up period is less than the intervention phase |  |
|  |  | 2 | Fellow up period is equal to or greater than the intervention phase |  |

|  |  |  |  |  |
| --- | --- | --- | --- | --- |
| 7. | Follow-up Rate | 0 | Less than 70% completion | 0.73 |
|  |  | 1 | 70-84.9% completion |  |
|  |  | 2 | 85-100% completion |  |
|  |  |  |  |  |
| 8. | Collaterals | 0 | No collateral verification of participant self-report | 0.54 |
|  |  | 1 | Collaterals interviewed |  |
|  |  |  |  |  |
| 9. | Dosage | 0 | 0-2 sessions (the full intervention) or no discussion of number of sessions received | 0.54 |
|  |  | 1 | 3-9 sessions |  |
|  |  | 2 | 10+ sessions |  |
|  |  |  |  |  |
| 10. | Dropouts | 0 | No discussion or enumeration of dropouts | 0.54 |
|  |  | 1 | Intervention dropout enumerated and/or discussed |  |
|  |  |  |  |  |
| 11. | Analyses | 0 | No statistical analyses conducted or clearly inappropriate analyses | 0.31 |
|  |  | 1 | Appropriate statistical analyses presented |  |
|  |  |  |  |  |
| 12. | Multisite | 0 | Single site study | 0.42 |
|  |  | 1 | Parallel replication at two or more sites |  |
|  |  |  |  |  |
| 13. | Cultural, linguistic and/or developmental adaptations | 0 | Adaptations not reported or discussed | 0.69 |
|  |  | 1 | Adaptations reported and/or discussed |  |
|  |  |  |  |  |
| 14. | Generalizability | 0 | No discussion of generalizability of findings | 0.54 |
|  |  | 1 | Discussion of generalizability of findings |  |

Sources: Auslander et al.,2012; Cabassa et al., 2016; Miller, 1995

Scores may range from “0” (low) to “18” (high).

^a^ Percent agreement of investigator ratings for each domain
